# Calcium entry units (CEUs): perspectives in skeletal muscle function and disease

**DOI:** 10.1007/s10974-020-09586-3

**Published:** 2020-08-18

**Authors:** Feliciano Protasi, Laura Pietrangelo, Simona Boncompagni

**Affiliations:** 1 CAST, Center for Advanced Studies and Technology, University G. d’Annunzio of Chieti-Pescara, 66100 Chieti, Italy; 2 DMSI, Department of Medicine and Aging Sciences, University G. d’Annunzio of Chieti-Pescara, 66100 Chieti, Italy; 3 DNICS, Department of Neuroscience, Imaging and Clinical Sciences, University G. d’Annunzio of Chieti-Pescara, 66100 Chieti, Italy

**Keywords:** Sarcoplasmic reticulum (SR), Store-operated Ca^2+^ entry (SOCE), Transverse tubules (TT), Tubular aggregate myopathy (TAM)

## Abstract

In the last decades the term Store-operated Ca^2+^ entry (SOCE) has been used in the scientific literature to describe an ubiquitous cellular mechanism that allows recovery of calcium (Ca^2+^) from the extracellular space. SOCE is triggered by a reduction of Ca^2+^ content (i.e. depletion) in intracellular stores, i.e. endoplasmic or sarcoplasmic reticulum (ER and SR). In skeletal muscle the mechanism is primarily mediated by a physical interaction between stromal interaction molecule-1 (STIM1), a Ca^2+^ sensor located in the SR membrane, and ORAI1, a Ca^2+^-permeable channel of external membranes, located in transverse tubules (TTs), the invaginations of the plasma membrane (PM) deputed to propagation of action potentials. It is generally accepted that in skeletal muscle SOCE is important to limit muscle fatigue during repetitive stimulation. We recently discovered that exercise promotes the assembly of new intracellular junctions that contains colocalized STIM1 and ORAI1, and that the presence of these new junctions increases Ca^2+^ entry via ORAI1, while improving fatigue resistance during repetitive stimulation. Based on these findings we named these new junctions Ca^2+^ Entry Units (CEUs). CEUs are dynamic organelles that assemble during muscle activity and disassemble during recovery thanks to the plasticity of the SR (containing STIM1) and the elongation/retraction of TTs (bearing ORAI1). Interestingly, similar structures described as *SR stacks* were previously reported in different mouse models carrying mutations in proteins involved in Ca^2+^ handling (calsequestrin-null mice; triadin and junctin null mice, etc.) or associated to microtubules (MAP6 knockout mice). Mutations in *Stim1* and *Orai1* (and calsequestrin-1) genes have been associated to tubular aggregate myopathy (TAM), a muscular disease characterized by: (a) muscle pain, cramping, or weakness that begins in childhood and worsens over time, and (b) the presence of large accumulations of ordered SR tubes (tubular aggregates, TAs) that do not contain myofibrils, mitochondria, nor TTs. Interestingly, TAs are also present in fast twitch muscle fibers of ageing mice. Several important issues remain un-answered: (a) the molecular mechanisms and signals that trigger the remodeling of membranes and the functional activation of SOCE during exercise are unclear; and (b) how dysfunctional SOCE and/or mutations in *Stim1, Orai1* and calsequestrin (*Casq1)* genes lead to the formation of tubular aggregates (TAs) in aging and disease deserve investigation.

## Store-operated Ca^2+^ entry in skeletal muscle function and disease

### ***Store-operated Ca***^***2***+^***entry (SOCE)***

Store-operated Ca^2+^ entry (SOCE), first described as *capacitative Ca*^*2*+^ *entry* in non-excitable cells (Putney 1986), is one of main Ca^2+^ entry mechanisms in cells that allow the replenishment of intracellular stores, i.e. the endoplasmic reticulum (ER). After the initial discovery, it was shown that Ca^2+^ release-activated calcium (CRAC) channels were involved in the mechanism (Hoth and Penner 1992). Then, the molecular bases of SOCE remained elusive for more than a decade, until the molecular players of SOCE were finally identified: (a) stromal interaction molecule-1 (STIM1), a Ca^2+^ sensor in the ER membrane (Liou et al. 2005; Roos et al. 2005; Zhang et al. 2005) and (b) ORAI1, a Ca^2+^-permeable channel (CRAC) of the plasma membrane (PM) (Prakriya et al. 2006; Vig et al. 2006; Wu et al. 2006; Yeromin et al. 2006). It is now becoming clear that SOCE may contribute to a variety of Ca^2+^ dependent cell functions such as gene transcription, migration, proliferation, differentiation, and also muscle contractility (Courjaret and Machaca 2012; Lopez et al. 2020; Moccia et al. 2012; Moccia et al. 2016; Ong et al. 2019; Potier et al. 2009; Stiber et al. 2008).

The mechanism that leads to activation of SOCE was first studied in non-excitable cells by light microscopy using fluorescent-tagged proteins. Briefly, upon Ca^2+^ depletion of internal stores, Ca^2+^ dissociates from the N-terminal EF-hand domains of STIM1 located in the lumen of the ER (Liou et al. 2005). This leads to conformational changes with consequent dimerization of STIM1 in the ER membrane and translocation towards the PM enabling STIM1 to interact and activate ORAI1 Ca^2+^ channels. This STIM1-ORAI1 interaction occurs in discrete junctional regions, defined as *puncta*, between ER and PM (Luik et al. 2006; Wu et al. 2006; Zhang et al. 2005). In this mechanism, a movement of the STIM1-bearing ER membranes toward the surface membrane is required for obtaining a direct close opposition of ER Ca^2+^ stores and PM containing ORAI1. As in a feedback loop, Ca^2+^ entry via STIM1-activated ORAI1 channels is used to then replenish depleted ER Ca^2+^ stores, which was the initial signal for its activation.

### ***Ca***^***2***+^***handling and SOCE in skeletal muscle***

In muscle, excitation contraction (EC) coupling, the mechanism linking the depolarization of PM to SR Ca^2+^ release, has evolved into different mechanisms: *Ca*^*2*+^*-induced Ca*^*2*+^ *release* (CICR), which uses external Ca^2+^ to induce release from internal stores in cardiac and smooth muscle (Fabiato 1983; Protasi et al. 1996; Sun et al. 1995); and *mechanical coupling* of skeletal muscle (Chandler et al. 1976; Rios et al. 1991; Schneider 1994). The term *mechanical* is used because the voltage-gated L-type Ca^2+^ channels of TTs, i.e. CaV 1.1 also known as dihydropyridine receptors (DHPRs), are directly linked to the Ca^2+^ release channels of the SR, the ryanodine receptors type-1 (RYR1) (Block et al. 1988; Paolini et al. 2004; Protasi 2002). This direct link allows formation of DHPR-tetrads, in which 4 DHPRs associate to the four subunits of RYR1 (Block et al. 1988; Protasi et al. 1998).

As mechanical EC coupling is extracellular Ca^2+^ independent, the role of extracellular Ca^2+^ in skeletal muscle function and contractility has been neglected for many years. Evidence for a Ca^2+^ entry pathway independent from DHPRs was reported in the 90′s (Gissel and Clausen 1999), before the first evidence of a Ca^2+^ entry pathway activated by depletion of Ca^2+^ in the SR was collected in mouse skeletal muscle fibers (Kurebayashi and Ogawa 2001). Later, the fact that muscle performance was reduced under conditions that inhibit SOCE supported the idea that SOCE contributes to contractility, especially during repetitive stimulation (Pan et al. 2014; Wei-Lapierre et al. 2013). Initially, it has been suggested that SOCE in muscle was mediated by coupling of Ca^2+^ release channels such as RYR or inositol-trisphosphate (INSP3) receptors in the SR membrane with transient receptor potential cation channels (TRPC) in the PM (Launikonis et al. 2003; Pan et al. 2002; Rosenberg et al. 2004). The discovery of SOCE being mediated by STIM1 and ORAI1 in non-excitable cells (Feske et al. 2006; Roos et al. 2005) helped a few years later the identification of STIM1 and ORAI1 as the main players of SOCE also in skeletal muscle fibers (Lyfenko and Dirksen 2008). In support of this finding, the demonstration that SOCE is abolished in mice lacking STIM1 (Stiber et al. 2008) and in muscle fibers from dominant-negative and muscle-specific *Orai1*- knockout mice (Carrell et al. 2016; Wei-Lapierre et al. 2013).

There is now general agreement regarding the fact that STIM1/ORAI1-dependant Ca^2+^-entry via SOCE increases force generation during prolonged stimulation (Boncompagni et al. 2017; Michelucci et al. 2019, 2020; Thornton et al. 2011). Indeed, removal of extracellular Ca^2+^ and addition of SOCE channel inhibitors (e.g. 2-APB, BTP-2) reduced the ability of skeletal muscle to maintain contractile force during prolonged stimulation (Boncompagni et al. 2017). Furthermore, strong evidence has been collected in latest years also regarding the role that SOCE plays in long-term functions of muscle such as differentiation, development and growth. Indeed, both STIM1-ORAI1 expression and SOCE activity are enhanced during differentiation of myotubes (Darbellay et al. 2009; Stiber et al. 2008), while their knockout reduces muscle mass in mice (Li et al. 2012; Wei-Lapierre et al. 2013).

It has been speculated that some features of STIM1-ORAI1 interaction in skeletal fibers must be different from that occurring in non-excitable cells, mainly because of differences in the time-course of its activation. In non-muscle cells the process, from ER store depletion to ORAI1 channel activation, takes tens of seconds (Wu et al. 2006), while in skeletal muscle some authors have proposed that Ca^2+^ influx can be activated very rapidly (< 1 s) following Ca^2+^ store depletion (Edwards et al. 2010; Launikonis and Rios 2007). This led some authors to conclude that the rapid activation of SOCE in muscle could be only explained by the presence of pre-formed SR-TT junctions promoting a preferential and fast access of STIM1 to ORAI1.

A few years later it has been suggested that muscle may contain two functionally distinct pools of STIM1: (a) one pool that mediates rapid SOCE activation located at the triad; and (b) a reserve pool at the I-band that would produce graded SOCE following store depletion (Stiber et al. 2008). Darbellay and colleagues discovered a *Stim1* splice variant highly expressed in skeletal muscle, *Stim1*-long, that could indeed account for rapid SOCE activation at the triad where the TTs contain ORAI1 (Darbellay et al. 2009, 2011).

### SOCE in muscle dysfunction and disease

As Ca^2+^ ions represent a versatile second messenger that controls a variety of cellular functions, dysregulation of Ca^2+^ homeostasis is often associated to dysfunction and skeletal muscle diseases (Avila-Medina et al. 2018; MacLennan 2000). Depressed or accelerated SOCE has been associated to several distinct forms of muscle dysfunction, i.e. weakness in aging (Brotto 2011; Pan et al. 2014; Thornton et al. 2011), muscular dystrophy in *mdx* mice (Zhao et al. 2012; Goonasekera et al. 2014; Onopiuk et al. 2015), and oversensitivity to heat in malignant hyperthermia (Yarotskyy and Dirksen 2012; Yarotskyy et al. 2013).

SOCE dysfunction can be directly caused by mutations in genes encoding for STIM1 and ORAI1, which can cause loss- or gain-of-function in the two proteins. *Loss-of-function* mutations in the genes encoding *STIM1* and *ORAI1* have been linked to patients affected by a severe combined immunodeficiency, which is often combined with severe myopathies (Feske 2009; Feske et al. 2010; McCarl et al. 2009; Picard et al. 2009). McCarl et al. 2009 and Lian et al. 2018 indeed reported loss-of-function mutations in *ORAI1* causing type I predominance and atrophic (or completely missing) type II fibers, while loss-of-function mutations in *STIM1* have been also associated to severe muscle hypotonia (Fuchs et al. 2012; Picard et al. 2009).

On the other hand, *gain-of-function* mutations in the same two genes have been linked to three different diseases: Stormorken and York Platelet syndromes (Bohm and Laporte 2018; Borsani et al. 2018; Lacruz and Feske 2015; Nesin et al. 2014; Markello et al. 2015; Misceo et al. 2014; Morin et al. 2020) and to a rare form of myopathy known as tubular aggregate myopathy or TAM (Bohm et al. 2013, 2014; Bulla et al. 2019; Endo et al. 2015; Okuma et al. 2016; Walter et al. 2015).

Tubular aggregates (TAs) are unusual membranous structures, which represent an important indicator of human myopathies, and that have been also found in aging mice, where they preferentially assemble in fast twitch fibers (Boncompagni et al. 2012a; Chevessier et al. 2005). It is generally accepted that TAs originate mostly from SR membranes, while TTs and mitochondria seems to be excluded (Boncompagni et al. 2012a; Chevessier et al. 2005; Salviati et al. 1985; Vielhaber et al. 2001). The clinical spectrum of TAM varies: from asymptomatic, to slowly progressive limb weakness, to muscle pain and cramping, to joint deformities in the arms and legs (Engel et al. 1970; Jain et al. 2008; Pierobon-Bormioli et al. 1985; Stormorken et al. 1985). A murine model harboring the most common TAM/Stormorken syndrome mutation in the *Stim1* gene (R304W) has been recently characterized and manifested a multi-systemic phenotype, but no formation of TAs was reported (Silva-Rojas et al. 2019).

Recently, also calsequestrin-1 (CASQ1), a protein that acts as the main Ca^2+^ buffer in the SR (MacLennan et al. 1983) and that plays a central role in skeletal EC coupling, has been proposed to modulate SOCE by a retrograde signal that inhibits STIM1 aggregation (Shin et al. 2003; Wang et al. 2015; Zhang et al. 2016). Three novel mutations in the *CASQ1* gene have been identified in patients affected by TAM (Barone et al. 2017): Laporte and colleagues suggested that mutations in the *CASQ1* gene impair its polymerization and cause TAM (Bohm et al. 2018).

## Calcium Entry Units, newly characterized intracellular SR/TT junctions that promote STIM1-ORAI1 colocalization and enhance Store-operated Ca^2+^ entry

### Searching for SOCE-sites in muscle

Different electron microscopy (EM) studies proposed ER-PM junctions that form following depletion of intracellular stores as the SOCE sites (Orci et al. 2009; Wu et al. 2006). Perni and colleagues using EM combined with freeze-fracture preparations studied the localization of both STIM1 and ORAI1 in HEK-293 cells: in this study overexpression of *Stim1* and/or *Orai1* induced formation of stacks of ER cisternae joined together by electron-dense small linkers, with ORAI1 clustered in specialized domains of the PM. It is worth emphasizing that overexpression of *Stim1* in this study induced the formation PM invaginations (structures similar to primitive TTs of muscle) associated to ER stacks (Perni et al. 2015).

Skeletal muscle fibers are the largest mammalian cells resulting from the fusion of progenitor cells (known as myoblasts) into myotubes. While myotubes grow in size, the nuclei that are initially placed approximately in the center of myotubes, move toward the periphery to leave space for the contractile, metabolic, and Ca^2+^ handling machineries (Engel and Franzini Armstrong 1994). When finally mature, adult skeletal fibers appear very densely packed with organelles that apparently occupy pre-determined positions: (a) myofibrils aligned transversally to create the striated appearance of skeletal fibers visible in histology; (b) most mitochondria placed at the I band in proximity of Z lines; (c) triads, the sites of excitation–contraction (EC) coupling, placed at the I-A band junction of sarcomeres, often closely associated to mitochondria (Bolanos et al. 2008; Boncompagni et al. 2009, 2020; Franzini-Armstrong and Boncompagni 2011; Rossi et al. 2009, 2011). Triads, also known as Calcium Release Units (CRUs), are sites where TTs (which carry the action potentials in the fiber interior) and the SR (that contains releasable Ca^2+^) come in close contact with each other. CRUs are the specialized intracellular junctions allowing the *mechanical coupling* between DHPRs, or CaV1.1 L-type Ca^2+^ channel, with the SR Ca^2+^ release channels, the RYR1 (Franzini-Armstrong and Protasi 1997; Schneider and Chandler 1973; Schneider 1994).

Activation of SOCE requires an interaction between STIM1, the Ca^2+^ sensor of SR membranes, and ORAI1, a Ca^2+^ release activated channel (CRAC) placed in external membranes, either the PM of its invaginations, i.e. the TTs (Liou et al. 2005; Prakriya et al. 2006; Roos et al. 2005; Vig et al. 2006; Wu et al. 2006; Yeromin et al. 2006; Zhang et al. 2005). As detailed in “Store-operated Ca^2+^ entry in skeletal muscle function and disease”, it has been suggested that the rapid kinetics of SOCE activation in skeletal muscle (Edwards et al. 2010; Launikonis and Rios 2007; Launikonis et al. 2009) would require a preformed site of interaction between STIM1 and ORAI1 (Dirksen 2009). As in triad junctions TTs and SR are already associated to each other to mediate EC coupling, this site would in principle represent the perfect location for SOCE (Dirksen 2009; Launikonis and Rios 2007; Launikonis et al. 2009). Indeed, in triads TTs (external membranes which may contain ORAI1) and the SR (which contains STIM1) are already in close proximity and could in principle allow a prompt and direct molecular interaction between STIM1 and ORAI1 during the rapid SOCE activation in muscle.

This hypothesis, though, was not supported by direct experimental evidence and it should be acknowledged that some peculiar features of triad junctions may represent a limit for proper STIM1-ORAI1 interaction. The junctional SR-TT gap at the triad is a quite crowded space filled by the large cytoplasmic domains of RYRs (known as feet). In the triadic junction RYR-feet are in direct contact with one another, corner-to-corner, forming regular arrays (Ferguson et al. 1984; Paolini et al. 2004), and are mechanically coupled with DHPR*-tetrads* that are placed across the junctional gap in the TT membrane (Protasi et al. 1998; Protasi 2002). Additionally, many other proteins such as junctophilins, FKBP12, triadin, junctin, mitsugumin, STAC3, etc., modulate EC coupling and participate in the assembly and maintenance of the triad junction (Al-Qusairi and Laporte 2011; Boncompagni et al. 2012b; Ito et al. 2001; Nelson et al. 2013; Protasi et al. 1997; Tang et al. 2004). Hence, the complex macromolecular machinery contained in triads may result in only limited opportunity for movement of STIM1 oligomers to recruit ORAI1 channels that are located across the triadic gap in the junctional portion of TTs.

The issue raised above suggests the possibility that SOCE may occur at sites different from the triads. Recently published manuscripts, discussed in the following sections of this Chapter, suggest that the most likely site for SOCE may indeed not be the triad (Boncompagni et al. 2017, 2018; Michelucci et al. 2018, 2020).

### Remodeling of internal membranes and increased STIM1-ORAI1 colocalization induced by exercise

**Fig. 1:**
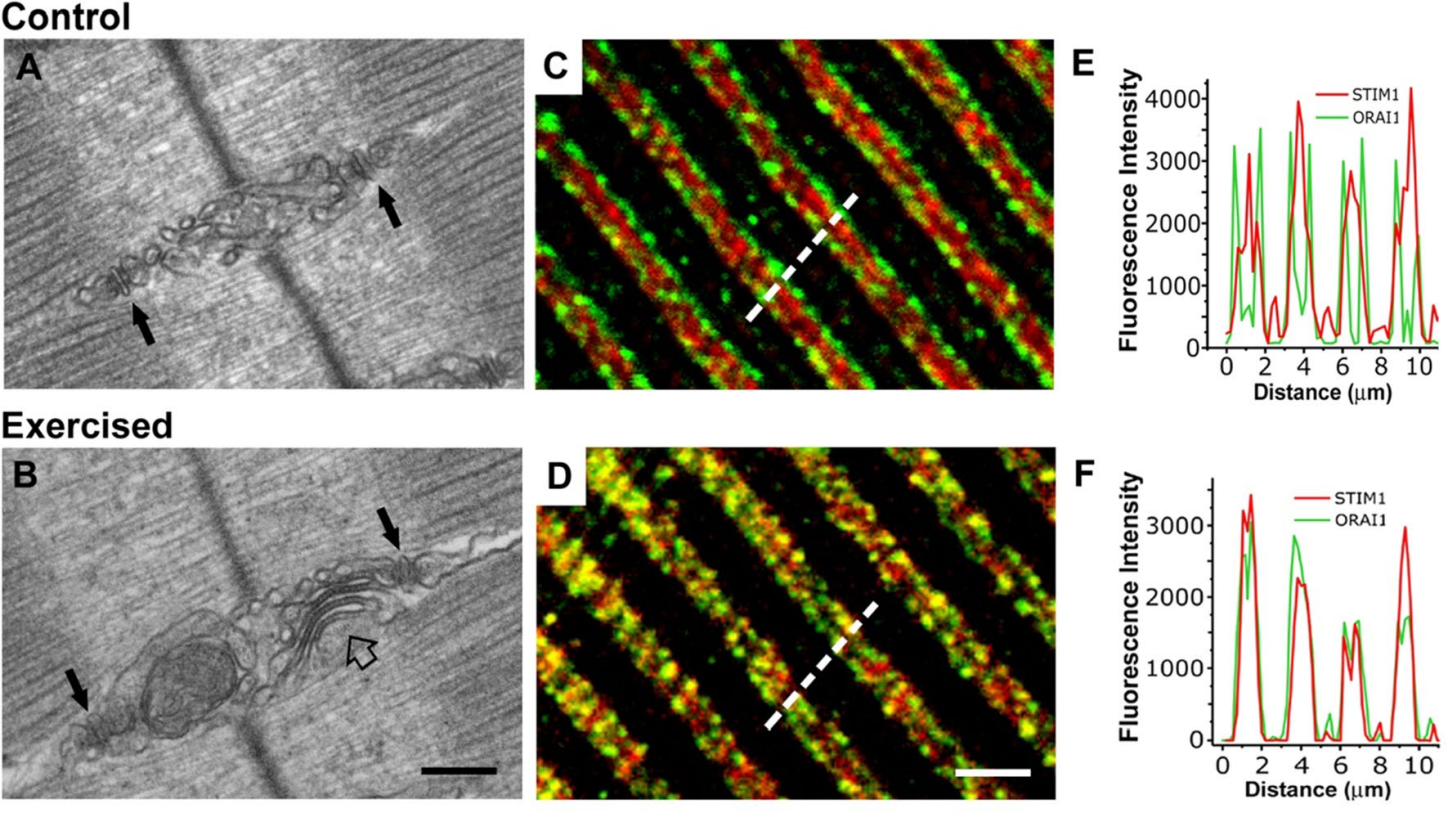
**Exercise results in remodeling of internal membranes and increase in STIM1-ORAI1 colocalization**. **a** and **b** Representative electron-micrographs obtained from EDL fibers from control (panel **a**) mice and exercised (panel **b**) mice: black arrows point to triads, while the empty arrow in B points to an SR stack at the I band. **c** and **d** Representative immunofluorescence images obtained from EDL fibers double-labeled for STIM1-ORAI1 from control (panel **c**) mice and exercised (panel **d**) mice. **e** and **f** Fluorescence intensity profile obtained along 5 µm distance (see dashed line) in fibers from control mice and exercised mice. In control conditions (**c** and **e**) STIM1 is preferentially localized at the I band of sarcomeres, with a low degree of colocalization with ORAI1, while following exercise (**d** and **f**) their degree of colocalization is significantly increased. Scale bar: 0.2 µm (**a**, **b**); 2 µm (**c**, **d**)

Recent evidence indicates that one hour of incremental running on a treadmill determines a striking remodelling of internal membranes of the sarcomere in EDL muscle of mice (Fig. 1). The SR at the I band in control conditions forms a complex network that in longitudinal sections appear as vesicles and convoluted tubes packed in multiple layers between Z line and triad (Fig. 1a). Following exercise, though, these membranes may rearrange in stacks of flat and parallel cisternae, which are formed by 2 or more elements (Fig. 1b). In these SR stacks the individual cisternae appear bridged together by several small electron dense strands which span a junctional gap of about 8 nm (Boncompagni et al. 2017). These stacks resemble those that form in non-muscle cells overexpressing *Stim1* (Orci et al. 2009; Perni et al. 2015), and present features that allow to clearly distinguish them from triadic junctions (CRUs). First, the electron densities in triads, i.e. RYR-feet, are significantly larger in size than the strands in SR stacks: indeed RYR-feet require a junctional space of about 12 nm to be allocated. Second, the cisternae in SR stacks do not contain the electron-dense polymer (representing CASQ) that is present in the terminal cisternae of triads (Boncompagni et al. 2012b; Franzini-Armstrong and Protasi 1997; Franzini-Armstrong et al. 1987).

Boncompagni and colleagues (Boncompagni et al. 2017, 2018) showed that the remodeling of membranes at the I band during exercise, not only involved the SR, but also the TTs which extended from triads into the I band towards the stacks of SR. Finally, immunolabeling for confocal microscopy (and immonogold for EM) pointed to increased colocalization between STIM1 and ORAI1 following treadmill running (Fig. 1c–f). Before exercise STIM1 appeared preferentially localized at the I band of sarcomere (Fig. 1c and e), with a low degree of colocalization with (both RYR1 and) ORAI1, which display an almost exclusive triadic positioning. On the other hand, following exercise the degree of colocalization between the two proteins was significantly increased (Fig. 1d and f) due to migration of part of the ORAI1 signal toward the I band containing STIM1. The easier explanation for this result would be that the relocation of ORAI1 may be the direct consequence of the TT elongation into the I band. One additional interpretation of these findings would be that the interaction between STIM1 and ORAI1 (required for SOCE activation) would be reduced in control conditions due to low degree of colocalization, while it should be facilitated and augmented following exercise.

### Exercise increases resistance to fatigue and ORAI1-dependent Ca^2+^ entry

Structural remodeling of intracellular membranes following exercise was accompanied by significant changes in the functional output of EDL muscles.

1. Extensor digitorum longus (EDL) muscles from pre-exercised animals were more resistant to fatigue than control muscles during repetitive stimulation in presence of 2.5 mM external Ca^2+^, but this augmented fatigue resistance was abolished in condition that prevented Ca^2+^ entry, i.e. Ca^2+^ free extracellular solution, or presence of SOCE blockers (Boncompagni et al. 2017) such as BTP-2 and 2-APB (Bootman et al. 2002; Zitt et al. 2004).
2. The functional implications of the exercise-induced remodeling of SR and elongation of TT were dissected more in depth in Michelucci et al. 2019 studying single fibers isolated from flexor digitorum brevis (FDB) using Mn^2+^ quenching of Fura-2 fluorescence, the state-of-the-art technique to measure entry of divalent cations from the extracellular space. Results of those experiments pointed to a significantly augmented entry of Mn^2+^ both following depletion of intracellular stores (+ depletion) and even in absence of depletion (- depletion) in FDB fibers of pre-exercised animals compared to controls. These findings were complemented by the demonstration that this Ca^2+^ entry pathway was dependent on presence of ORAI1, as the exercise-induced adaptations were absent in two different models of *Orai1*-knockout: (a) dominant-negative *Orai1* transgenic mice (Wei-Lapierre et al. 2013) and (b) tamoxifen-inducible *Orai1* knockout mice (Carrell et al. 2016). Finally, Michelucci et al. 2019 also determined how the Ca^2+^ entry induced by the remodeling of SR and TT membranes at the I band increased Ca^2+^ transients, resting Ca^2+^, and contractile force during repetitive stimulation.

In conclusion, the increased rate of Mn^2+^ quench in FDB fibers containing the SR-TT remodeling suggests that the new junctions that form during exercise function as *Ca*^*2*+^ *Entry Units* (CEUs) to support SOCE during repetitive muscle activity. This conclusion is also reinforced by immunofluorescence and immunogold labeling in Boncompagni et al. 2017, pointing to increased colocalization of STIM1 and ORAI1 following exercise.

### Ca^***2***+^ Entry Units are constitutively assembled in mice lacking calsequestrin-1

Calsequestrin is a high-capacity Ca^2+^-binding protein located in the lumen of the SR terminal cisternae (Campbell et al. 1983; Yano and Zarain-Herzberg 1994). There is a general consensus that calsequestrin is not only important for the SR ability to store Ca^2+^, but also for modulating Ca^2+^ release from RYRs (Beard et al. 2002, 2005; Ikemoto et al. 1989). Structural studies suggested that calsequestrin is indeed in the right location to control the activity of the Ca^2+^ release channels (Franzini-Armstrong et al. 1987; Saito et al. 1984). Recent evidence suggests that CASQ1 also modulates SOCE (Barone et al. 2017; Shin et al. 2003; Wang et al. 2015; Zhao et al. 2010).

**Fig. 2:**
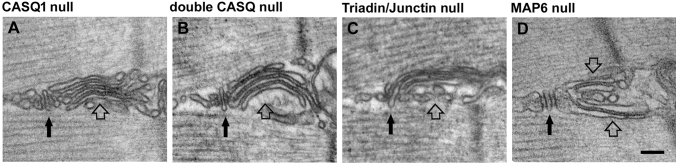
**Exercise-like SR remodeling is also found in animal models with defective Ca**^**2+**^ **handling**. Structures virtually identical to SR stacks formed during exercise (pointed by empty arrows) are often found also in mouse models carrying ablation of proteins involved in Ca^2+^ handling at the triad (pointed by black arrows), such as calsequestrin, triadin and junctin, and in *Map6* knockout mice. Scale bar: 0.1 μm

Skeletal muscles from mice lacking calsequestrin-1 (*Casq1*-null) exhibit reduced SR Ca^2+^ content and undergo rapid and deep SR Ca^2+^ depletion (the key signal to activate SOCE) during sustained, high-frequency stimulation (Paolini et al. 2007; Canato et al. 2010). In the first paper describing the structure of the EC coupling system of these knockout mice (Paolini et al. 2007), the presence of SR stacks was overseen. Later the presence of SR stacks was reported in fibers from mice lacking CASQ1 and 2 (Boncompagni et al. 2012a; see “Exercise-like sarcotubular remodeling is also found in animal models with defective Ca^2+^ handling”). Very recently Michelucci and colleagues reported that: (a) in skeletal muscle from mice lacking calsequestrin-1, CEUs are constitutively assembled in EDL and FDB muscles (Michelucci et al. 2020; an SR stack in *Casq1*-null muscle is shown in Fig. 2a); (b) the presence of CEUs correlated with enhanced constitutive- and store-operated Ca^2+^ entry; and (c) the recovery of Ca^2+^ ions via SOCE served to promote enhanced maintenance of peak Ca^2+^ transient amplitude, and increased maintenance of contractile force during repetitive, high-frequency stimulation.

### T-tubule plasticity controls the assembly of functional Calcium Entry Units (CEUs)

Data in Michelucci et al. 2019 indicate that T-tubules elongation into the I band during exercise is required for increased Mn^2+^ quench. Indeed, this work contains a time-course study in which assembly and disassembly of CEUs was studied < 1 h, 6 h and 24 h after a single bout of treadmill running: a) TTs retracts from SR stacks within 6 h following exercise; b) SR stacks disappear within 24 h. The fact that SOCE function is enhanced when TTs extension and contacts with SR stacks are increased, but returns to control levels once TTs retract from the I band, points to a central role of TTs in the assembly of functional CEUs. The mechanism underlying TT elongation and the reason of a higher plasticity of TT vs. SR stacks remain elusive and deserve further investigation (see “Open Questions and Future Directions” for more detail).

## Exercise-like sarcotubular remodeling is also found in animal models with defective Ca^2+^ handling

The SR, the organelle with the major impact on Ca^2+^ homeostasis of skeletal muscle, is a convoluted compartment composed by tubules and cisternae, which forms a continuous network surrounding each myofibril (Rossi and Dirksen 2006). It is essentially composed of two continuous domains: junctional and longitudinal SR. The junctional SR flanks a central T-tubule to form the triad, also known as CRU, a structure deputed to Ca^2+^ accumulation and release during EC coupling thanks to the presence of CASQ and RYRs. The longitudinal SR, on the other hand, surrounds both A and I bands and it is important for Ca^2+^ re-uptake as it contains SERCA pumps (Flucher et al. 1993). Next to the I band, the longitudinal SR is formed by multiple layers (instead of a single layer as at the A band), possibly because at the I band more space is available as at rest contractile filaments are not overlapped. The proteins involved in the maturation of both longitudinal and junctional SR, and how these proteins are retained in different and specialized domains depending on their role/function, have been extensively reviewed (Rossi et al. 2008). Al-Qusairi and Laporte provided also additional insight about the role that proteins such as caveolin 3, amphiphysin 2, dysferlin, mitsugumins, junctophilins, etc. may play in T-tubule biogenesis, triad formation and/or maintenance, and their implication in muscle disorders (Al-Qusairi and Laporte 2011).

The longitudinal SR at the I band is the one that provides the membranes that undergo exercise-induced remodeling resulting in formation of SR stacks (see “Calcium Entry Units, newly characterized intracellular SR/TT junctions that promote STIM1-ORAI1 colocalization and enhance Store-operated Ca^2+^ entry”). Plasticity of the sarcotubular system during differentiation and maturation of muscle fibers has been studied (Flucher et al. 1993). Modification of EC coupling membranes during aging and disease have been also reported in muscle from humans and mice (Boncompagni et al. 2006; Chevessier et al. 2004; Lee and Noguchi 2016; Pietrangelo et al. 2015). Less is known, though, about the sarcotubular plasticity in healthy adult muscle. Reviewing the existing literature, though, we realized that SR stacks virtually identical to those formed during exercise in adult mice (see “Calcium Entry Units, newly characterized intracellular SR/TT junctions that promote STIM1-ORAI1 colocalization and enhance Store-operated Ca^2+^ entry”) were also noted in mouse models carrying either ablation of proteins involved in EC coupling and in a mouse with a null mutation to a microtubule associate protein (Fig. 2). What follows are brief descriptions of those studies:

### Ko et al. (2011)

Junctophilins (JPs) are a family of proteins that contribute to the formation of junctional membrane complexes linking the PM (or its invaginations) with the ER and SR in non-excitable and excitable cells (Takeshima et al. 2000). JPs provides a structural foundation for functional crosstalk between the cell surface and intracellular stores. JPs are the likely candidates for the docking of the junctional SR to TT in triads of skeletal muscle (Ito et al. 2001; Takeshima et al. 2000). Hirata and colleagues in 2006 reported that knockdown of *Jp1* and *Jp2* (two different isoforms) caused a drastic reduction on SOCE activation in C2C12 cells (Hirata et al. 2006). The same group of authors a few year later, reported the effect of inducing transient knockdown of *Jps* in adult muscle fibers, concluding that reduced expression of JP1 and JP2 leads to abnormal junctional membrane structure and defective Ca^2+^ signaling (reduced SR Ca^2+^ store and defective operation of SOCE). In the EM images published by Ko et al. 2011, SR stacks virtually identical to those formed during exercise in Boncompagni et al. 2017 are clearly visible. In this paper SR stacks were described as *irregular triad junctions*.

### Boncompagni et al. (2012a)

Two *Casq* isoforms are expressed during skeletal muscle development: a skeletal and a cardiac isoform, respectively *Casq1* and *Casq2* (Fliegel et al. 1987; Scott et al. 1988). In slow muscle, the CASQ2 protein is the most abundant isoform in fetal and neonatal stages, whereas in adults it accounts for 25–50% of the total CASQ present (Damiani et al. 1990). In fast twitch fibers, CASQ2 disappears at two-four weeks post-natally in rodents (Sacchetto et al. 1993). In Boncompagni et al. 2012a the presence of SR stacks was reported in muscle fibers of 4–6 month old double *Casq*-null mice: in that paper no specific analysis of TTs (the second element required for the assembly of functional CEUs) was performed at the time. An SR stack in double *Casq*-null muscles in shown in Fig. 2b. Now we know that also EDL and FDB fibers of *Casq*1-null mice contain a great amount of CEUs and their presence correlates with enhanced SOCE (Michelucci et al. 2020; see also “Calcium Entry Units, newly characterized intracellular SR/TT junctions that promote STIM1-ORAI1 colocalization and enhance Store-operated Ca^2+^ entry”).

### Boncompagni et al. (2012b)

Junctin and triadin (Caswell et al. 1991; Jones et al. 1995; Knudson et al. 1993) are two SR proteins believed to anchor CASQ to the terminal cisternae. Ultrastructural evidence for CASQ connection to SR membranes comes from the observations of strands linking the junctional SR membrane to the electron-dense content of terminal cisternae, in deep-etch studies of vertebrates muscles (Franzini-Armstrong et al. 1987).

Triadin and junctin are also believed to modulate the activity of RYR1 Ca^2+^ release channels (Guo and Campbell 1995) facilitating the cross-communication between CASQ and RYR both in cardiac and skeletal muscle (Beard et al. 2002; Gyorke et al. 2004; Zhang et al. 1997). Several lines of evidence have suggested that triadin is also an important regulator of the myoplasmic Ca^2+^ homeostasis in skeletal muscle (Eltit et al. 2010; Fodor et al. 2008; Shen et al. 2007). Boncompagni et al. 2012b reported ho ablation of triadin and junctin caused evident alterations of the triad structure, i.e. reduction in size of terminal cisternae and loss of proper position of CASQ away from RYRs. Though, this work also reported that: (a) fibers from triadin-null and triadin/junctin double null muscles contain unusual accumulation of flat SR cisternae at the I band; and (b) SR cisternae are separated by small densities that are clearly different from feet. An SR stack in double triadin/junctin null fiber in shown in Fig. 2c. The SR cisternae displayed in Boncompagni et al. 2012b are virtually identical to the SR stack assembled in wild type (WT) adult mice following exercise (Boncompagni et al. 2017) and contain the small densities that bridge the gap between the different SR elements. The presence of SR stacks in triadin-null and double triadin/junctin null muscles was accompanied by functional alteration: SR Ca^2+^ content, peak of Ca^2+^ transient, and rate of Mn^2+^ quench were all reduced.

### Valle et al. (2016)

Valle and colleagues investigated the functional role of calsequestrin-2 (CASQ2) in slow- and fast-twitch skeletal muscles using knockout mice (Knollmann et al. 2006; Valle et al. 2016). CASQ2 is expressed throughout life in slow-twitch muscle fibers, but only in the first months of life in fast-twitch fibers. The authors claim that CASQ2 knockout results in increased expression of CASQ1, and no functional defects were detected in either EDL or Soleus muscles. Interestingly, the authors also report the presence of SR stacks which appear virtually identical to those that form in WT muscle following exercise in Boncompagni et al. 2017.

### Sebastien et al. (2018)

Skeletal muscle fibers are characterized by an extraordinary internal organization, which is at the basis of their efficient muscle contraction. One important player in this intracellular organization, achieved during differentiation and maturation of adult fibers, is the microtubule network (Khairallah et al. 2012; Oddoux et al. 2013; Prins et al. 2009; Sebastien et al. 2018; Sparrow and Schock 2009). The microtubule network is dynamic and is regulated by many microtubule-associated proteins (MAPs). Sébastien and colleagues studied the role of the MAP6 protein in skeletal muscle organization and function using a *Map6* knockout mouse. *Map6* isoforms are known to stabilize microtubules in vitro against different challenges (Bosc et al. 1996; Denarier et al. 1998; Margolis et al. 1990). *Map6* deletion in mice affected microtubule organization and determined SR structural abnormalities, identified by EM, which include defects in triads and formation of extensive stacks of SR flat cisternae, again resembling those forming in muscle of WT type mice following treadmill exercise (Boncompagni et al. 2017). An SR stack in a *Map6* knockout fiber in shown in Fig. 2d. Structural modifications in Map6 knockout fibers were associated to defective EC coupling, i.e. reduced Ca^2+^ release causing reduced force output. No direct measurements of SOCE were performed in this study.

## Open Questions and Future Directions

The role of extracellular Ca^2+^ in skeletal muscle has been neglected for many years, possibly due to fact that EC coupling in skeletal muscle is mechanical and independent on Ca^2+^ entry (Brum et al. 1987; Brum and Rios 1987; Schneider and Chandler 1973; Schneider 1994). Though, over the past two decades the importance of Ca^2+^ entry for muscle function has gained renewed attention. On one side SOCE, the Ca^2+^ entry pathway extensively discussed in this manuscript and many reviews (Avila-Medina et al. 2018; Lopez et al. 2020; Michelucci et al. 2019; Ong et al. 2019), is activated by a decrease of Ca^2+^ in SR stores, i.e. depletion (Lyfenko and Dirksen 2008). On the other side, a pathway known as excitation-coupled Ca^2+^ entry (ECCE), independent of SR store depletion, also received considerable attention in the first decade of this millennium. ECCE is based on a retrograde signal from RYR1 to CaV1.1, the two main players in EC coupling, which activates a slow Ca^2+^ entry pathway, not required for EC coupling itself (Cherednichenko et al. 2004, 2008; Hurne et al. 2005; Yang et al. 2007).

The exercise-induced remodeling of the sarcotubular system in CEUs containing pre-localized STIM1 and ORAI1 which enhance constitutive and store-operated Ca^2+^ entry, is bringing new light upon the short-term plasticity of skeletal muscle fibers to external demands, in this case exercise. However, several important issues remain un-answered.

### The basic mechanism that initiates/promotes assembly of SR stacks and CEUs remains elusive

SOCE is activated by depletion of intracellular stores, a signal that leads to oligomerization of STIM1 and recruitment/activation of ORAI1 and allows Ca^2+^ entry (Luik et al. 2006; Wu et al. 2006; Zhang et al. 2005). However, the mechanism underlying the morphological changes in SR/TT membranes which results in assembly of CEUs and increased STIM1-ORAI1 colocalization in muscle fibers requires further investigation. The collection of papers discussed in “Exercise-like sarcotubular remodeling is also found in animal models with defective Ca^2+^ handling” suggests a possible common mechanism leading to assembly of SR stacks (i.e. reduced amount of Ca^2+^ stored in the SR, with consequent depletion). Unfortunately, in those papers a detailed analysis of TTs, the second element required for the assembly of functional CEUs, is missing. Hence, a final conclusion about the presence of fully assembled CEUs cannot be drawn. See Other Unresolved Issues (*Is assembly of SR stacks in different mouse models underlined by a common mechanism?*) for additional detail. To understand more about the molecular mechanisms underlying membrane remodeling future efforts should be focused in studying the role that proteins known to be involved in TT biogenesis and membrane-bending, such as bridging integrator-1 (BIN1) also known as amphiphysin-II, caveolin-3 (CAV3), myotubularin-1 (MTM1), junctophilins (JPs) (Al-Qusairi and Laporte 2011; Dowling et al. 2009; Lee et al. 2002; Takeshima et al. 2015), etc., may play in this process.

### The role that changes in intracellular micro-environment may play in membrane remodeling has not been explored

It is well known that during exercise several intracellular parameters change in muscle fibers (Allen 2004; Allen et al. 2008a, b; Allen and Trajanovska 2012; Lamb 2002, Lamb 2009; Lamb and Westerblad 2011; Westerblad and Allen 2011):

- reduction in pH, due to production of carbon dioxide during aerobic metabolism and to glycolysis that may produce excessive pyruvate and accumulation of lactic acid.
- Transient accumulation of reactive species of oxygen and nitrogen (ROS and RNS), now widely recognized as signaling molecules in muscle function.
- Decrease in ATP/ADP ratio and increase in levels of inorganic phosphate, whose concentration can increase rapidly during intense fatigue.
- Increase in temperature, due to generation of heat during aerobic metabolism and break down of ATP.
- Reduction in partial pressure of oxygen (hypoxia), due to increased 0_2_ consumption during aerobic ATP production.
- Repetitive shortening and relaxation of contractile elements, which is sensed by mechano-sensors in contractile filaments and cytoskeleton (Piazzesi et al. 2018; Ward et al. 2014).

Interestingly, STIM1 has been proposed to be a multipurpose stress transducer activated by diverse stimuli, beside Ca^2+^ store depletion (Soboloff et al. 2012): (a) ROS were shown to induce STIM1 aggregation, translocation to ER–PM junctions and activation of ORAI1 channels without store depletion (Hawkins et al. 2010); (b) increased temperature from 37 °C to 41 °C was shown to trigger STIM1 activation independently of Ca^2+^ store depletion (Mancarella et al. 2011a; Xiao et al. 2011); (c) hypoxic stress and decreased ATP levels cause Ca^2+^ store depletion and activation of STIM1 proteins, even if coupling to ORAI1 channels seems prevented in this case (Mancarella et al. 2011b).

In view of the above, it is possible that STIM1 may act as a sensor for other stimuli, such as pH, ROS, temperature and/or mechanical modification of membrane compartments.

### How mutation in STIM1, ORAI1, and CASQ1 genes (and dysfunctional SOCE in aging) lead to the formation of tubular aggregates is not clear

Tubular aggregates (Tas), that preferentially assemble in fast twitch fibers (Engel et al. 1970), are accumulation of ordered SR tubes, often or regular size, which represent an important indicator of human muscle disorders, including TA myopathy (TAM). TAM begins in childhood and worsen over time with leg muscles most often affected. Affected individuals may have an unusual walking style (gait) or difficulty running, climbing stairs, or getting up from a squatting position. TAM has been linked to mutations is STIM1 and ORAI1 genes (Bohm et al. 2013, 2014; Nesin et al. 2014). However, the mechanism that from mutations in those proteins leads to assembly of TAs is not understood. Bohm and colleagues suggested that mutations in *STIM1* can induce its constitutive clustering, and that constitutively active STIM1 seems the major cause of a steadily elevated myoplasmic Ca^2+^ (Bohm et al. 2013). TAM may also result in STIM1-independent activation of CRAC channels due to dominant mutations in ORAI1 (Nesin et al. 2014) and to suppression of the slow Ca^2+^-dependent inactivation of the CRAC channel (Endo et al. 2015).

Recently, TAs were also found in patients with missense mutations in the *CASQ1* gene. CASQ1 mutants showed reduced ability to store Ca^2+^ and a reduced inhibitory effect on SOCE (Barone et al. 2017). Also Bohm and colleagues identified *CASQ1* as the third TAM gene (Bohm et al. 2018). Clinical, histological, genetic, and functional data supported the finding that *CASQ1* mutations significantly impair its polymerization and depolymerization and result in aggregation of STIM1, thus providing a pathological link between STIM1- and CASQ1-related TAM (Bohm et al. 2018).

TAs, though, have been also found in muscles of male aging mice, almost exclusively in fast twitch fibers (Boncompagni et al. 2012a; Chevessier et al. 2004). Aging muscle is characterized by muscle weakness and depressed SOCE (Brotto 2011; Thornton et al. 2011; Zhao et al. 2008), but why TAs preferentially assemble in fast twitch fibers and in muscle of male mice during aging is unclear. One hypothesis is that accumulation of TAs in muscle fibers from male mice may be influenced by sex hormones (Baltgalvis et al. 2010) and possibly mediated by oxidative stress levels, which is usually higher in males than in females (Borras et al. 2003). Regarding the fiber type specificity: based on the demonstration that TAs represent sites of Ca^2+^ accumulation, probably slow twitch fibers do not develop TAs because of their greater amount of mitochondria which can help in buffering excess of Ca^2+^ (Chevessier et al. 2004). Other possibilities are that slow twitch fibers contain a lower amount of SR membranes (the membranes recruited to form TAs), and that they are better protected against cellular stress thanks to higher oxidative metabolism (Chevessier et al. 2004).

### Other unresolved issues

- *CEUs increase in size/number during exercise also in slow twitch fibers?* Presence of SR stacks, and exercise dependent assembly of CEUs, has been noticed and studied in EDL muscles, which are mainly constituted by fast twitch fibers. In EDL, CEUs are small and few in control animals, and increase in frequency and size following exercise. Will this be the case also in slow twitch fibers, or this increased assembly of SOCE-sites occurs only in fast twitch fibers, which cycle Ca^2+^ at a higher rate during EC coupling and re-uptake?
- *What about CEU assembly in long-term training?* Boncompagni et al. 2017 and Michelucci et al. 2019 analyzed the effect of a single bout of treadmill running. However, what happens in long-term training has not been investigated. Studying the effects of training could disclose different scenarios: (a) constitutive presence of CEUs in muscle fibers from trained animals; (b) greater expression of *Stim1* and *Orai1* that could mediate a faster and more effective response to exercise.
- *Do* STIM1-short *and* STIM1-long *play different roles in the exercise induced assembly of Ca*^*2*+^ *Entry Units?* Stiber and colleagues proposed two functionally distinct pools of STIM1, one at the triad that mediates rapid SOCE and another one that serves as a reserve pool within the longitudinal SR at the I-band which could mediate recruitment of additional SOCE when needed (Stiber et al. 2008). Darbellay and colleagues discovered a *Stim1* splice variant highly expressed in skeletal muscle (*Stim1*-long) which should be involved in permanent clusters with ORAI1 at the triad (Darbellay et al. 2009, 2011). In this picture, graded recruitment of additional SOCE activity could be mediated by STIM1S, which indeed appears distributed throughout the longitudinal SR at the I band, in the perfect position to contribute to the assembly of CEUs during exercise.
- *Is assembly of SR stacks in different mouse models underlined by a common mechanism?* Reviewing the papers reporting the presence of SR stacks in “Exercise-like sarcotubular remodeling is also found in animal models with defective Ca^2+^ handling” (Boncompagni et al. 2012a; Michelucci et al. 2020; Ko et al. 2011; Sebastien et al. 2018; Valle et al. 2016) it is tempting to hypothesize a common mechanism that underlies their formation, i.e. a reduced SR Ca^2+^ store content which may predispose to easier depletions of the SR during repetitive muscle activity. However, this hypothesis is supported only by some, but not all, of these papers:
- In *Casq1*-null and double *Casq*-null fibers, which contain a great amount of SR stacks (Boncompagni et al. 2012a; Michelucci et al. 2020), SR Ca^2+^ content is severely reduced, SR undergoes severe depletion when repetitively stimulated, and rate of Mn^2+^ quench is reduced (Paolini et al. 2007, 2011; Michelucci et al. 2020).
- The presence of SR stacks in triadin-null and double triadin/junctin-null muscles was accompanied by reduced (i) SR Ca^2+^ content, (ii) peak of Ca^2+^ transient, and (iii) rate of Mn^2+^ quench (Boncompagni et al. 2012b).
- Ko et al. 2011. showed that reduced expression of JP1 and JP2 leads to modification of SR structure (with formation of *irregular triad junctions*, in our eyes clearly SR stacks) accompanied by defective Ca^2+^ signaling (reduced SR Ca^2+^ store and defective operation of SOCE).
- On the other hand the following two papers do not contain experimental evidence to support the hypothesis of a common mechanism underlying formation of SR stacks:
- In the paper of Valle and colleagues, in skeletal muscle fibers lacking CASQ2 no defects in SR Ca^2+^ content were reported (Valle et al. 2016).
- In *Map6* knockout mice, the authors evaluated the SR content, but did not report a decrease in the amount of stored Ca^2+^ (only alteration in EC coupling, i.e. reduced Ca^2+^ release causing reduced force output, but no measurements of SOCE were performed) (Sebastien et al. 2018).

## Closing remarks

### Summary

The present manuscript describes recent findings regarding the remodeling of SR and TT membranes during exercise, which may have a significant impact on SOCE activity and skeletal muscle function:

1. exercise induces remodeling of intracellular membranes, resulting in assembly of new SR-TT junctions at the I band formed by SR stacks and extension of triadic TTs (Boncompagni et al. 2017, 2018);
2. these new SR-TT junctions promote STIM1-ORAI1 colocalization, and increase resistance of muscles during repetitive stimulation in presence of external Ca^2+^ (Boncompagni et al. 2017, 2018);
3. the presence of these junctions correlates with increased rate of Mn^2+^ quench and augmented amplitude of Ca^2+^ transients (Michelucci et al. 2019);
4. the elongation of TTs into the I band during muscle activity and its retraction following recovery controls the assembly of functional CEUs (Michelucci et al. 2019);
5. CEUs are constitutively assembled in *Casq*-null fibers, which are functionally defective, because of a significantly reduced SR Ca^2+^ content and tendency to undergo marked SR Ca^2+^ depletion during high-frequency stimulation (Canato et al. 2010; Michelucci et al. 2020);
6. the presence of SR stacks have been reported previously in several other mouse models (junctophilin and triadin/junctin-null mice, etc.) (Boncompagni et al. 2012a, b; Ko et al. 2011; Sebastien et al. 2018; Valle et al. 2016).

### Assembly of Ca^2+^Entry Units and up-regulation of SOCE during exercise may represent an acute adaptation of muscle aiming to maintain proper intracellular Ca^2+^ levels

The mechanisms that underlie muscle fatigue are very complex (Allen et al. 2008a). One of the contributing factors is the loss of Ca^2+^ in the extracellular space during repetitive muscle stimulation, as this would cause a reduction in releasable Ca^2+^ from the SR during EC coupling (Allen et al. 2008b). In this scenario the assembly during exercise of specific SR-TT junctions deputed to recover external Ca^2+^ (i.e. CEUs) may represent a positive adaptation of muscle, triggered by SR depletion, aiming to create a preferential pathway to recover extracellular Ca^2+^, replenish the SR, and possibly delay the onset of (or reduce) muscle fatigue. This hypothesis is supported by findings in *Casq1*-null muscle, where the constitutive presence of CEUs in *Casq1*-null muscle is likely an adaptation of fibers to the chronic reduced amount of Ca^2+^ in the SR. With this in mind we could hypothesize that CEUs creates an open door for prompt access to extracellular Ca^2+^ supply when internal Ca^2+^ stores cannot provide sufficient ions from muscle function.

## Abbreviations

CASQ1: Calsequestrin-1
CEU: Calcium entry unit
CRU: Ca^2^^+^ release unit
EDL: Extensor digitorum longus
EC Coupling: Excitation–contraction coupling
EM: Electron microscopy
ER: Endoplasmic reticulum
FDB: Flexor digitorum brevis
PM: Plasma membrane
SOCE: Store operated Ca^2^^+^ entry
SR: Sarcoplasmic reticulum
STIM1: Stromal interaction molecule-1
TA: Tubular aggregate
TAM: TA myopathy
TT: Transverse tubule
